# Pharmacologic cholinesterase inhibition improves survival in acetaminophen-induced acute liver failure in the mouse

**DOI:** 10.1186/1471-230X-14-148

**Published:** 2014-08-19

**Authors:** Niels Steinebrunner, Carolin Mogler, Spiros Vittas, Birgit Hoyler, Catharina Sandig, Wolfgang Stremmel, Christoph Eisenbach

**Affiliations:** 1 Department of Gastroenterology, Hepatology, Intoxications and Infectious Diseases, Heidelberg University Hospital, Im Neuenheimer Feld 410, 69120 Heidelberg, Germany; 2 Department of Pathology, Heidelberg University Hospital, Im Neuenheimer Feld 220/221, 69120 Heidelberg, Germany; 3 Department of Endocrinology and Clinical Chemistry, Heidelberg University Hospital, Im Neuenheimer Feld 410, 69120 Heidelberg, Germany; 4 Mailing address: Heidelberg University Hospital, Im Neuenheimer Feld 410, 69120 Heidelberg, Germany

**Keywords:** Acute liver failure, Acetaminophen, Cholinergic anti-inflammatory pathway, Cholinesterase inhibition

## Abstract

**Background:**

Acetaminophen (APAP) is one of the most widely used analgesic and antipyretic pharmaceutical substances in the world and accounts for most cases of drug induced liver injury resulting in acute liver failure. Acute liver failure initiates a sterile inflammatory response with release of cytokines and innate immune cell infiltration in the liver. This study investigates, whether pharmacologic acetylcholinesterase inhibition with neostigmine diminishes liver damage in acute liver failure via the cholinergic anti-inflammatory pathway.

**Methods:**

Acute liver failure was induced in BALB/c mice by a toxic dose of acetaminophen (APAP). Neostigmine and/or N-acetyl-cysteine (NAC) were applied therapeutically at set time points and the survival was investigated. Liver damage was assessed by serum parameters, histopathology and serum cytokine assays 12 h after initiation of acute liver failure.

**Results:**

Serum parameters, histopathology and serum cytokine assays showed pronounced features of acute liver failure 12 h after application of acetaminophen (APAP). Neostigmine treatment led to significant reduction of serum liver enzymes (LDH (47,147 ± 12,726 IU/l vs. 15,822 ± 10,629 IU/l, p = 0.0014) and ALT (18,048 ± 4,287 IU/l vs. 7,585 ± 5,336 IU/l, p = 0.0013), APAP-alone-treated mice vs. APAP + neostigmine-treated mice), inflammatory cytokine levels (IL-1β (147 ± 19 vs. 110 ± 25, p = 0.0138) and TNF-α (184 ± 23 vs. 130 ± 33, p = 0.0086), APAP-alone-treated mice vs. APAP + neostigmine-treated mice) and histopathological signs of damage.

Animals treated with NAC in combination with the peripheral cholinesterase inhibitor neostigmine showed prolonged survival and improved outcome.

**Conclusions:**

Neostigmine is an acetylcholinesterase inhibitor that ameliorates the effects of APAP-induced acute liver failure in the mouse and therefore may provide new treatment options for affected patients.

## Background

Acetaminophen (APAP) is one of the most commonly used pharmaceuticals in the world. It has a well-established record of safety and efficacy. However, taken in overdoses, APAP causes severe hepatic necrosis frequently leading to acute liver failure (ALF). APAP poisoning accounts for more than 30,000 hospital admissions and approximately 500 deaths every year in the U.S.A. alone [1-6]. With limited therapeutic options, besides the application of N-acetyl-cysteine (NAC), there is a need for further therapeutic alternatives to improve outcome and prevent death or orthotopic liver transplantation in affected patients [7-10]. APAP-induced ALF is a sterile inflammatory condition, with local and systemic inflammatory responses mediated by the release of pro-inflammatory cytokines from innate immune cells (e.g. neutrophils and Kupffer cells) and activation and migration of macrophages into the liver [11]. The cholinergic anti-inflammatory pathway responds to ongoing inflammation through the vagus nerve and nicotinic acetylcholine receptors (nAChRs) expressed by cytokine-producing cells, such as macrophages, neutrophils, dendritic cells, histiocytes, Kupffer cells and mastocytes [12-16]. The parasympathetic neurotransmitter acetylcholine is released and binds to the α7 subunit of the nAChR to prevent the unbalanced overproduction of inflammatory mediators, such as IL-1β and TNF-α [12,17,18].

The aim of the current study was to analyze the role of the acetylcholinesterase inhibitor neostigmine in modulation of APAP-induced acute liver failure via increasing the levels of acetylcholine and stimulation of the cholinergic anti-inflammatory pathway.

## Methods

### Reagents

Neostigmine was purchased from Actavis (Munich, Germany), acetaminophen (APAP) from Fresenius Kabi (Bad Homburg, Germany) and N-acetyl-cysteine (NAC) from CSC Pharmaceuticals (Bisamberg, Austria).

### Animals

Male BALB/c mice (Charles River Laboratories, Sulzfeld, Germany) at 10 weeks of age were used in all experiments. The animals received humane care and were kept on a 12-hour light/dark cycle in a temperature-controlled room, with free access to food and water. The protocol was approved by the Animal Care and Use Committee of the University of Heidelberg.

### Animal model and experimental groups

Acute liver failure was induced by intraperitoneal (i.p.) injections of APAP (600 mg/kg) after overnight food deprivation. Subsequently, the animals in the treatment group received an i.p. injection of the acetylcholinesterase inhibitor neostigmine (80 μg/kg) either 1 hour before or 1 hour after application of APAP as indicated, followed by successive applications of neostigmine after 7, 12 and 24 hours. Control mice received analogous volumes of saline. Dosing of APAP and neostigmine were based on earlier studies [19-21]. For assessment of liver damage, mice were sacrificed 12 hours after application of APAP and blood and tissue samples were harvested. In previous studies with similar dosing of APAP the peak level of histological and serological changes was reached after the selected time point [22-24]. Whole blood samples were allowed to clot and then centrifuged at 1000 g for 5 minutes. Serum was collected and stored at −80°C. Liver sections were fixed in 4% phosphate buffered formalin and embedded in paraffin for histological analysis.

In subsequent experiments mice were dosed with 750 mg/kg APAP and an additional application of 300 mg/kg (1.84 mmol/kg) NAC i.p. 2 hours thereafter. Furthermore, after intoxication with 750 mg/kg APAP, mice were either dosed with 75 mg/kg (0.46 mmol/kg) NAC i.p. after 2 hours, as the sole treatment, or along with neostigmine (80 μg/kg) i.p. after 2, 7, 12 and 24 hours [25-28].

For the survival experiments mice were monitored throughout the experimental period.

### Assays

Serum alanine aminotransferase (ALT), aspartate aminotransferase (AST) and lactate dehydrogenase (LDH) were measured in the Institute of Clinical and Laboratory Medicine at the University Hospital Heidelberg by standard procedures.

Serum was subjected to enzyme-linked immunosorbent assay (ELISA) for determination of IL-1β and TNF-α contents according to the manufacturers recommendations. ELISA kits were purchased from Quiagen (Gaithersburg, MD, USA).

### Histology

Livers were fixed in 4% buffered formalin and embedded in paraffin. Sections (3 μm in thickness) were cut and H & E staining was performed according to standard protocols. Slides were evaluated by an experienced liver pathologist (C.M.) blinded to the origin of the specimens with special regard to liver architecture, cellular changes, extent of necrosis (% of liver), and level of hemorrhage scored on a four-point scale (0–3; 0 none, 1 mild, 2 moderate, 3 severe). For the terminal deoxynucleotidyl transferase-mediated dUTP nick-end labeling (TUNEL) assay, sections of liver (3 μm in thickness) were stained with the ApopTag Apoptosis Detection Kit (Merck Millipore, Billerica, MA, USA) as described in the manufacturer’s instructions. TUNEL-positive cells were counted in 10 randomly selected microscopic fields (×200) per section and expressed as percentages of the total number of hepatocytes.

### Statistical analysis

Variables are expressed by mean and standard deviation. Statistical significance was evaluated using Student’s t-test or Mann-Whitney’s *U* test. The survival curve obtained by the Kaplan-Meier procedure was analyzed by log-rank test. A p value < 0.05 was considered statistically significant. Statistical analysis was performed using GraphPad Prism software (version 6.0, GraphPad Software, Inc., La Jolla, CA, USA).

## Results

### Cholinesterase inhibition with neostigmine improves survival in APAP-induced acute liver failure

**Figure 1:**
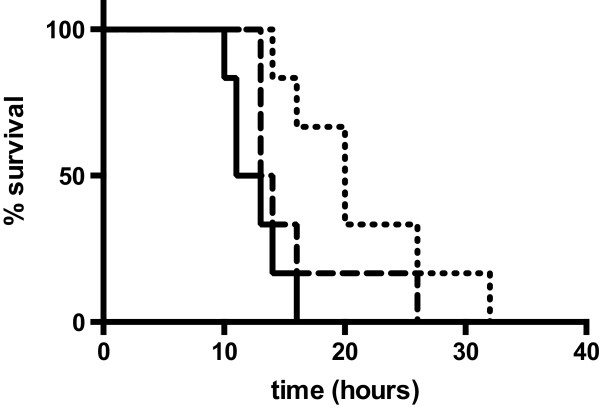
**Cholinesterase inhibition protects against acute liver failure (ALF) induced by acetaminophen (APAP).** All mice received 600 mg/kg APAP i.p.. Neostigmine-treated animals received 80 μg/kg neostigmine 1 hour prior to application of APAP and 7, 12 and 24 hours thereafter (dotted line, p_log-rank_ = 0.0046) or 1, 7, 12 and 24 hours after application of APAP (dashed line, p_log-rank_ = 0.1860). Control mice received solvent (0.9% NaCl) 1, 7, 12 and 24 hours after application of APAP (solid line; n = 6 for each group).

Animals treated with neostigmine (80 μg/kg) one hour before intoxication with APAP (600 mg/kg) and repeatedly 7 and 12 hours afterwards showed significantly improved survival compared to control animals (mean survival time in hours 21 ± 7 vs. 13 ± 2, p_*log-rank*_ = 0.0046). To assess a delayed application of neostigmine as a therapeutic option after application of APAP, animals were treated with neostigmine (80 μg/kg) 1 hour after intoxication with APAP and repeatedly 7 and 12 hours afterwards. Therapeutic treatment resulted in prolonged survival compared to control animals (mean survival time in hours 16 ± 5 vs. 13 ± 2, p_*log-rank*_ = 0.1860), which however failed to reach statistical significance (Figure 1).

### Cholinesterase inhibition with neostigmine improves hepatocellular damage in APAP-induced acute liver failure

**Figure 2:**
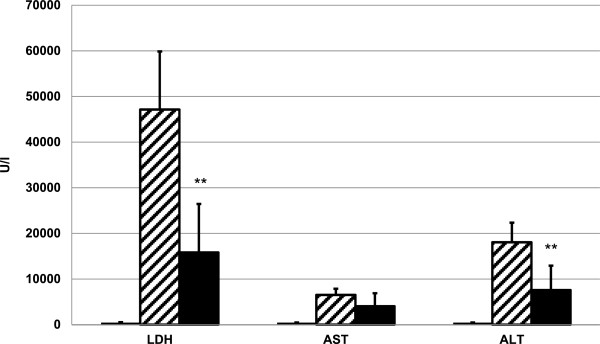
**Serum levels of lactate dehydrogenase (LDH), aspartate aminotransferase (AST) and alanine aminotransferase (ALT) after acetaminophen (APAP)-induced liver injury are reduced by cholinesterase inhibition.** 12 hours after application of 600 mg/kg APAP i.p., serum was collected from neostigmine-treated (80 μg/kg i.p. 1 and 7 hours after application of APAP) and control mice. LDH and serum-transaminases were measured (mean ± SD; *open bars*, control mice, *hatched bars*, APAP + solvent, *filled bars*, APAP + neostigmine; n = 6 for each group; **p < 0.01 APAP-alone-treated mice vs. APAP + neostigmine-treated mice).

Following intoxication with APAP (600 mg/kg) i.p., animals were either treated with neostigmine (80 μg/kg) 1 and 7 hours afterwards or vehicle was applied. Mice were sacrificed after 12 h and serum was collected and analyzed for enzyme activities indicating liver injury. Neostigmine alleviated APAP-induced liver damage as reflected by significant reduction in LDH (47,147 ± 12,726 IU/l vs. 15,822 ± 10,629 IU/l, p = 0.0014) and ALT (18,048 ± 4,287 IU/l vs. 7,585 ± 5,336 IU/l, p = 0.0013). There was still a considerable, although not significant decline in AST (6,522 ± 1,338 IU/l vs. 4,048 ± 2,828 IU/l, p = 0.1575) (Figure 2).

### Cholinesterase inhibition with neostigmine reduces histopathological liver damage and apoptosis in APAP-induced acute liver failure

**Figure 3:**
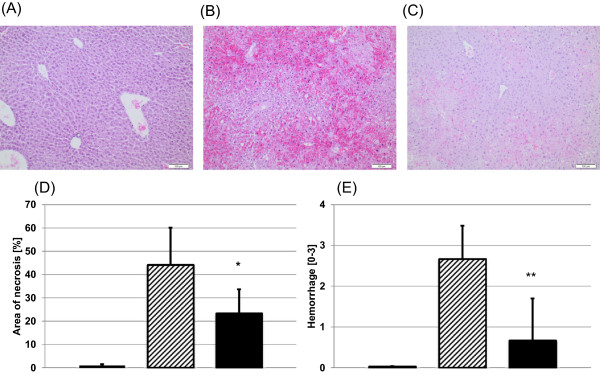
**Acetaminophen (APAP)-induced centrilobular necrosis in BALB/c mice is attenuated by the application of a cholinesterase inhibitor.** Liver injury was induced with 600 mg/kg APAP i.p.. After 1 and 7 hours 80 μg/kg neostigmine or vehicle control was applied. Liver tissue was harvested 12 hours after application of APAP and processed for histopathology. Representative H & E-stained liver sections (100× magnification), **(A)** control mice, **(B)** APAP + solvent, **(C)** APAP + neostigmine. **(D)** Area of necrosis (% of area). **(E)** Hemorrhage [0–3] (mean ± SD; *open bars*, control mice, *hatched bars*, APAP + solvent, *filled bars*, APAP + neostigmine; n = 6 for each group; *p < 0.05, **p < 0.01 APAP-alone-treated mice vs. APAP + neostigmine-treated mice).

**Figure 4:**
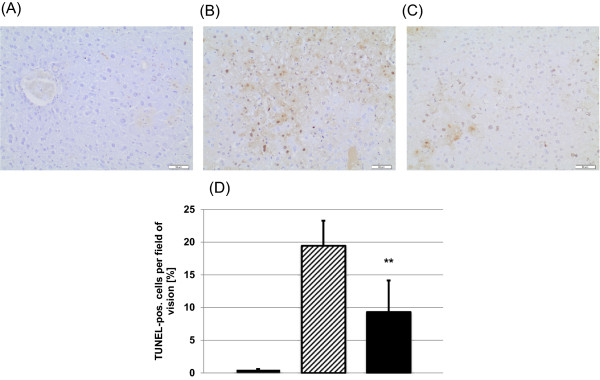
**Hepatic histological changes assessed by TUNEL assay in acetaminophen (APAP)-induced liver injury in BALB/c mice is attenuated by the application of a cholinesterase inhibitor.** Liver injury was induced with 600 mg/kg APAP i.p.. After 1 and 7 hours 80 μg/kg neostigmine or vehicle control was applied. Liver tissue was harvested 12 hours after application of APAP and processed for histopathology. Representative TUNEL-stained liver sections (200× magnification), **(A)** control mice, **(B)** APAP + solvent, **(C)** APAP + neostigmine. **(D)** TUNEL-positive cells per field of vision [%] (mean ± SD; *open bars*, control mice, *hatched bars*, APAP + solvent, *filled bars*, APAP + neostigmine; n = 6 for each group; **p < 0.01 APAP-alone-treated mice vs. APAP + neostigmine-treated mice).

In addition to serum enzyme levels we analyzed liver histopathology for signs of neostigmine-mediated protection from APAP-induced hepatotoxicity (Figure 3A-C). Mice that were administered neostigmine (80 μg/kg) 1 and 7 hours after intoxication with APAP (600 mg/kg) showed less extends of centrilobular necrosis than control mice (area of necrosis 44 ± 16% vs. 23 ± 10%, p = 0.0228) (Figure 3D). The extent of hemorrhage decreased in the neostigmine treatment group compared to control mice (3 ± 1 vs. 1 ± 1 on a scale from 0–3, p = 0.0065) (Figure 3E).To determine the extend of apoptosis we performed terminal deoxynucleotidyl transferase-mediated dUTP nick-end labeling (TUNEL) stainings of liver sections. Mice that were administered neostigmine (80 μg/kg) 1 and 7 hours after intoxication with APAP (600 mg/kg) showed less extends of DNA fragmentation than control mice (TUNEL-positive cells expressed as percentages of the total number of hepatocytes 19.5 ± 3.8% vs. 9.3 ± 4.8%, p < 0.0122) (Figure 4A-D).

### Cholinesterase inhibition with neostigmine reduces pro-inflammatory mediator generation in APAP-induced acute liver failure

**Figure 5:**
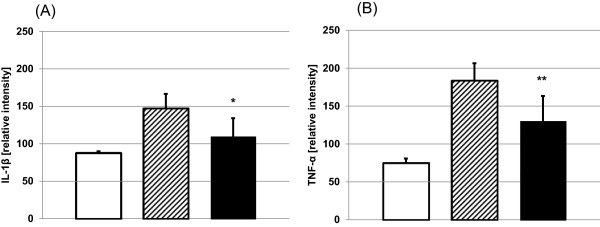
**Administration of neostigmine reduces serum cytokine levels.** 12 h after induction of acute liver failure by acetaminophen (600 mg/kg APAP i.p.), serum was collected from neostigmine-treated (80 μg/kg, 1 and 7 hours after application of APAP) and control mice. Cytokine concentrations of **(A)** IL-1β and **(B)** TNF-α were measured by enzyme-linked immunosorbent assay (mean ± SD; *open bars*, control mice, *hatched bars*, APAP + solvent, *filled bars*, APAP + neostigmine; n = 6 for each group; *p < 0.05, **p < 0.01 APAP-alone-treated mice vs. APAP + neostigmine-treated mice).

Treatment of mice with neostigmine (80 μg/kg) 1 and 7 hours after intoxication with APAP (600 mg/kg) significantly reduced IL-1β serum levels compared to control mice, which were applied vehicle (147 ± 19 vs. 110 ± 25, p = 0.0138). In two out of 6 neostigmine-treated animals, IL-1β even dropped below detection levels of the selected assay (Figure 5A). In line with a reduction of IL-1β, we could observe significantly lower levels of TNF-α (184 ± 23 vs. 130 ± 33, p = 0.0086) in neostigmine-treated mice (Figure 5B).

### NAC and neostigmine show an additive effect in the combined treatment of APAP-induced acute liver failure

Since N-acetyl-cysteine (NAC) is an established and frequently effective treatment for patients intoxicated with APAP, we evaluated an add-on therapeutic benefit of neostigmine.

**Figure 6:**
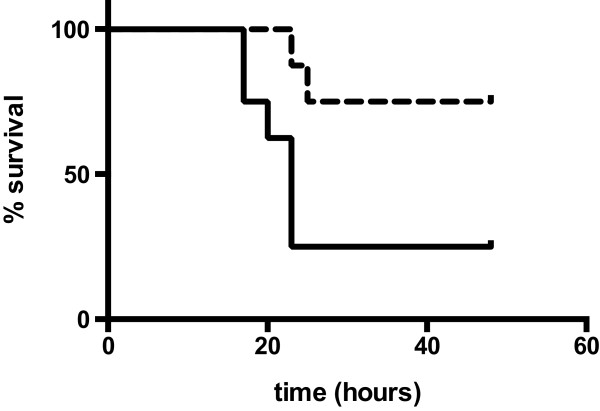
**Combined treatment with N-acetyl-cysteine (NAC) and neostigmine show an additive effect in acute liver failure (ALF) induced by acetaminophen (APAP).** All mice were treated with a dosing of 750 mg/kg APAP i.p. and a therapeutical treatment with 75 mg/kg NAC i.p. after 2 hours (solid line) or in a combined set-up with 75 mg/kg NAC after 2 hours and successive applications of neostigmine (80 μg/kg) i.p. after 2, 7, 12 and 24 hours (dashed line, p_log-rank_ = 0.0260; n = 8 for each group). Mice were monitored for 48 hours.

With a dosing of 750 mg/kg APAP and a therapeutical treatment with 300 mg/kg NAC after 2 hours all mice (4 of 4) survived a 48-hour observation period (data not shown), consistent with previously published data [25,27]. To assess the effect of combination treatment with neostigmine, NAC was applied at suboptimal doses [28]. All mice were treated with 750 mg/kg APAP and a therapeutical treatment with 75 mg/kg NAC after 2 hours or with 75 mg/kg NAC after 2 hours combined with successive applications of neostigmine (80 μg/kg) after 2, 7, 12 and 24 hours. In this experimental set-up neostigmine significantly (p_log-rank_ = 0.0260) prolonged survival and changed outcome. Specifically, during a 48-hour observation period, mortality was 6 of 8 for APAP plus NAC and 2 of 8 for APAP plus NAC plus neostigmine (Figure 6).

## Discussion

Acetaminophen (APAP) is one of the most frequently used analgesic and antipyretic agents in the world. The drug has an excellent safety profile in therapeutic doses, however ingestion of overdoses can have serious hepatotoxic effects and even induce fatal acute liver failure (ALF) [1-6].

APAP is metabolized predominantly by two pathways, comprising conjugation by sulfation and glucuronidation as well as oxidation to a reactive intermediate, N-acetyl-para-benzoquinone imine (NAPQI), which in turn is conjugated with glutathione to form non-toxic metabolites [29,30]. Ingestion of APAP in supra-therapeutic doses leads to excessive production of NAPQI, depletion of intracellular stocks of glutathione and covalent binding of NAPQI to cellular and mitochondrial proteins and thereby causing nuclear DNA damage [31,32]. A series of intracellular events, via mitochondrial membrane dysfunction and modulation of gene transcription factors, and activation of the innate immune system of the liver, culminates in centrilobular hepatic necrosis [33,34]. Necrotic cells release various damage-associated molecular pattern (DAMP) molecules, such as high-mobility group box 1 (HMGB1), heat-shock proteins and DNA fragments, that are ligands for toll-like receptors (TLRs) on macrophages and other cell types [35]. Upon activation by DAMP molecules, innate immune cells infiltrate the damaged area and trigger the release of cytokines and chemokines, such as TNF-α and IL-1β, which lead to a massive hepatic infiltration of leukocytes, thereby causing sterile tissue inflammation that further amplifies the liver damage [4,23,31,32,36,37]. This excessive inflammatory response resulting in systemic inflammation can be more injurious than the inciting event and may progress to shock, diffuse coagulation, multiple organ failure and death [6,15,22].

Interference in cytokine pathways can serve as an important target to limit inflammatory responses. After application of APAP, mice lacking TNF-α receptor as well as mice treated with anti-TNF-α antibodies showed a reduction in the neutrophil response and a concomitant significant attenuation of liver damage [38,39]. In addition, mice lacking IL-1β receptor, or neutralization of IL-1β in wild-type mice, resulted in significantly less APAP toxicity and decreased collateral damage from inflammation [40-43]. However, these same factors and other mediators, including IL-4, IL-6, IL-10 and IL-13 have also been associated with recruitment of monocytes for liver regeneration and tissue repair [44-46].

Cumulatively, these studies suggest that a complex series of immune reactions play an important role in mitigating the detrimental effects of a disproportionate inflammatory response while promoting local regeneration [25]. Therefore alterations in the balance of pro- and anti-inflammatory cytokine formation may contribute to the toxicity in APAP-induced liver failure.

An imminent regulator of the innate immune response is the cholinergic anti-inflammatory pathway. The central nervous system responds to inflammation via the vagus nerve by inhibiting the excessive release of inflammatory cytokines to balance the unfavorable effects of a disproportionate inflammatory response [23]. Efferent vagus neurons release acetylcholine, which binds to the nicotinic acetylcholine receptor subunit α7 (α7 nAChR) expressed on the cell membrane of macrophages and other cytokine secreting cells. Binding of acetylcholine to α7 nAChR inhibits release of pro-inflammatory cytokines [14,47]. The cholinergic anti-inflammatory pathway has been beneficial in experimental models of inflammation, including sepsis [12,18,19,48], inflammatory bowel disease [49] and pancreatitis [45].

Augmentation of the efferent vagus nerve can be achieved by direct electrical simulation, peripheral stimulation by selective agonists of α7 nAChR, such as GTS-21 [20,45], nicotine [46] or cholinesterase inhibition with physostigmine or neostigmine [19,38]. The acetylcholinesterase inhibitor neostigmine, which enhances cholinergic signaling by increasing acetylcholine levels, improved survival and reduced cytokine levels of TNF-α and IL-1β and attenuated neutrophil lung infiltration in a cecal ligation and puncture sepsis model [19].

In our experiments we could show that neostigmine reduces the hepatotoxic effects of APAP and improves survival. Neostigmine improved liver function and lowered the levels of the inflammatory cytokines TNF-α and IL-1β. The protective effect of neostigmine by reducing systemic inflammation through the cholinergic pathway may be responsible for the improved survival following application of APAP. A trend toward a protective effect was even observed when the first dose of neostigmine treatment was delayed for 1 hour after intoxication with APAP. However, probably due to the limited case number in our study, statistical significance at the 5% level was missed.

Since NAC is the cornerstone of clinical treatment of APAP intoxication, potential clinical use of neostigmine would likely be in combination with NAC. We could show that neostigmine in a combination with NAC prolonged survival and thus had an additive effect.

A limitation in the comparison of data of previous models of APAP-induced liver failure in rodents is the strain-dependent susceptibility to liver injury. For example C57Bl/6 mice exhibit attenuated toxicity after APAP challenge compared to BALB/c mice for reasons not yet fully identified [39,42,50,51]. Furthermore, unfasted and female mice are less susceptible to APAP-hepatotoxicity [21,28,52].

ALF is a multistep process that involves apoptosis, necrosis and necroapoptosis. After intoxication with APAP, hepatocyte apoptosis occurs in the early phase (3 – 5 hours) and shifts towards necrosis at later time points (10 – 15 hours) [48]. In ALF, induction of the energy-consuming process of apoptosis may lead to massive necrosis, once energy resources are exhausted [49,53,54]. This shift in cell death dynamics is reflected by cytokeratin 18 levels in the serum in patients with ALF and has recently been implemented in a cytokeratin 18-based modification of the Model for End-Stage Liver Disease (MELD) score with an improved prediction of survival [49]. Consistent with this concept we could show decreased induction of apoptosis in livers of neostigmine treated mice, which may in turn lead to decreased liver necrosis and may provide a rationale for neostigmine treatment.

Cholinesterase inhibition with neostigmine in humans seems feasible, as it has long been established for clinical use for other applications such as antagonization of muscle relaxants [55].

## Conclusions

In conclusion our findings point to a potential benefit of the cholinesterase inhibitor neostigmine in acute liver failure induced by APAP by modulation of unbalanced anti-inflammatory pathways. Further studies are needed to determine the exact role of the cholinergic system in acute liver failure and to assess cholinesterase inhibitors as a potential therapeutic option in affected patients.

## Abbreviations

ALF: Acute liver failure; ALT: Alanine aminotransferase; APAP: N-acetyl-para-amino-phenol, acetaminophen; AST: Aspartate aminotransferase; DAMP: Damage-associated molecular pattern; ELISA: Enzyme-linked immunosorbent assay; GTS-21: 3-(2, 4-dimethoxybenzylidene)-anabaseine; H & E: Hematoxylin and eosin; HMGB1: High-mobility group box-1; i.p.: Intraperitoneal; IL-1β: Interleukin-1 β; LDH: Lactate dehydrogenase; NAC: N-acetyl-cysteine; nAChR: Nicotinic acetylcholine receptor; NAPQI: N-acetyl-para-benzoquinone imine; TLR: Toll-like receptor; TNF-α: Tumor necrosis factor α; TUNEL: Terminal deoxynucleotidyl transferase-mediated dUTP nick-end labeling.

## Competing interests

The authors declare that they have no competing interests.

## Authors’ contributions

NS collected and analyzed experimental results, performed the statistical analysis and drafted the manuscript. CM, SV, BH and CS collected and analyzed experimental results and helped to draft the manuscript. WS helped to draft the manuscript. CE conceived of the study, participated in its design and coordination, analyzed experimental results and helped to draft the manuscript. All authors read and approved the final manuscript.

## Pre-publication history

The pre-publication history for this paper can be accessed here:

http://www.biomedcentral.com/1471-230X/14/148/prepub
